# Efficacy of autogenous tooth block for lateral ridge augmentation compared with autogenous bone block: A systematic review and meta-analysis

**DOI:** 10.1097/MD.0000000000035326

**Published:** 2023-09-29

**Authors:** Delin Guan, Ruimin Zhao, Yan Guo, Jianxue Li, Na Ma, Jiaming Gong

**Affiliations:** a The 940th Hospital of Joint Logistic Support Force of Chinese People’s Liberation Army, Department of Stomatology, Lanzhou City, China; b Quzhou Hospital Affiliated to Wenzhou Medical University (Quzhou People’s Hospital), Department of Stomatology, Quzhou City, China; c The 940th Hospital of Joint Logistic Support Force of Chinese People’s Liberation Army, Out-patient department, Lanzhou City, China.

**Keywords:** alveolar bone grafting, alveolar ridge augmentation, autogenous tooth, dental implants, systematic review, tooth extraction

## Abstract

**Background:**

Autogenous tooth block (ATB) has been used as an alternative material for bone regeneration, but its efficacy compared with autogenous bone block (ABB) remains uncertain. The aim of this systematic review was to investigate and compare the clinical and histological performance of ATB and ABB grafts in lateral alveolar ridge augmentation (LARA).

**Methods:**

Electronic retrieval of MEDLINE, Embase, Cochrane Library (CENTRAL), Scopus, Web of Science, China national knowledge infrastructure, Wanfang data, SinoMed, and manual searching until July 2023 were used to identify controlled clinical trials employing ATB grafts in LARA. The identified reports included at least one of the following outcome variables: ridge width gain, graft resorption, postoperative complications, histology, and histomorphometry. Weighted or mean differences (MD), relative risk, and corresponding 95% confidence intervals (CI) were calculated. Descriptive analysis was applied to the qualitative statistics. The protocol followed the preferred reporting items for systematic reviews and meta-analyses statement and was prospectively registered in PROSPERO (CRD42023399611).

**Results:**

Four controlled clinical trials with 77 participants each using ATB and ABB grafts were included. Meta-analysis indicated that ATB grafts resulted in greater bone width (MD = 1.31, 95% CI [0.92, 1.71], *P* < .00001) and less graft resorption (MD = −0.71, 95% CI [−1.22, −0.21], *P* = .005) than ABB grafts on LARA. There was no statistical difference in postoperative complications between ATB and ABB grafts (relative risk = 0.81, 95% CI [0.32, 2.04], *P* = .66). Furthermore, the ATB grafts exhibited positive replacement resorption with alveolar bone for favorable signs of new bone activity on histology and histomorphometry.

**Conclusion:**

Within the limitations of this study, ATB grafts could serve as an alternative material for ABB to support LARA. Further research with a longer follow-up period is required to verify these findings.

## 1. Introduction

Lateral alveolar ridge deficiency following tooth loss, trauma, or infection unfavorably affects the ideal placement of dental implants.^[1,2]^ Lateral alveolar ridge augmentation (LARA) is considered necessary and practical for simultaneous or early stage.^[3]^ Several techniques have been proposed to augment the alveolar ridge width, including guided bone regeneration, the split crest technique, autogenous bone grafting, and distraction osteogenesis.^[4,5]^

The use of autogenous bone grafts for LARA has been called the “gold standard” because of its optimal osteoinduction, osteoconduction, and osteogenic capabilities.^[6–8]^ It has been shown to achieve a favorable success rate even in severe alveolar ridge atrophy.^[8]^ Autogenous bone block (ABB), harvested from the retromolar region or ramus of the mandible, possesses excellent biological properties, and spatial preservation and is frequently used as a preferred option for LARA.^[8,9]^ Nevertheless, some shortcomings (e.g., limited bone volume, risk of donor complications, and unpredictable resorption) have limited the extensive use of ABB grafts.^[10,11]^

From an auxanological perspective, teeth and alveolar bone originate from neural crest cells and share similarities in their tissue components.^[12,13]^ Specifically, dentin has an inorganic content of 70% to 75%, organic content of 20%, and water content of 10%; alveolar bone corresponds to 65%, 25%, and 10%, respectively.^[14,15]^ Furthermore, the organic matrix of dentin is rich in many growth factors (e.g., bone morphogenetic protein, osteopontin, and glycoprotein), which regulate bone resorption and participate in osteogenesis and calcification.^[16–18]^ These characteristics drive replacement resorption and remodeling of dentin and alveolar bone during healing. Many studies^[19–21]^ have reported positive evidence for using crushed root particles in extraction sockets, maxillary sinus floor lifting, and peri-implant bone defects. In contrast, its application in ridge defects without vascularized bony walls is limited owing to its lack of rigidity.

**Figure 1.**
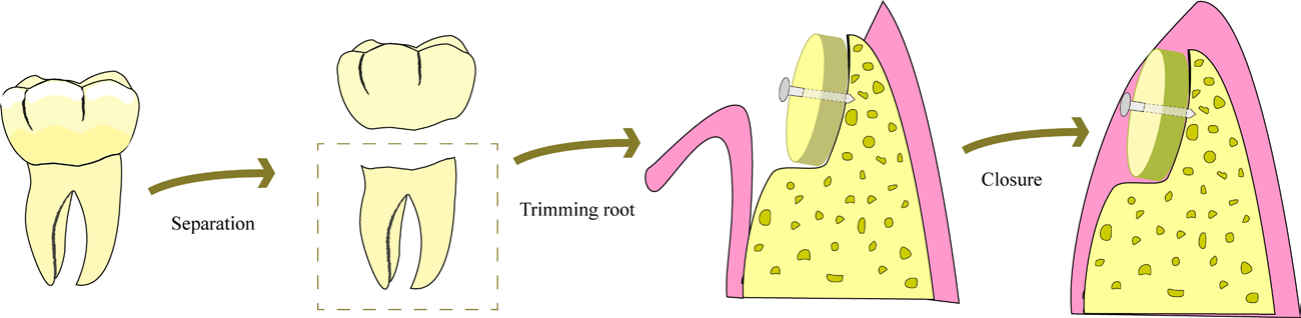
Diagram of the conversion of an autogenous tooth into an autogenous root block for lateral alveolar ridge augmentation.

Schwarz and colleagues^[22]^ introduced a chairside protocol for LARA using an autogenous tooth block (ATB). In this method, the ATB obtained by integral separation at the cementoenamel junction was modified to fit the alveolar ridge defect, allowing for easy fixation with microscrews and soft tissue closure (Fig. 1). Several animal and clinical trials^[22–25]^ have found that this technique performs well in dentin-bone integration and supports simultaneous or staged implant placement. Hence, the use of ATB grafts for LARA has been proposed as a potential alternative to ABB grafts; however, the inter-distinction still needs to be determined.

To our knowledge, there has yet to be a systematic review and meta-analysis of the quantitative assessment of LARA using ATB versus ABB grafts. Thus, this review aimed to assess the clinical and histological performance of ATB grafts in LARA compared to ABB grafts. We hypothesized that there would be no significant difference between the 2 graft types.

## 2. Materials and methods

### 2.1. Protocol development and focused question

The systematic review was conducted according to the preferred reporting items for systematic reviews and meta-analyses statement.^[26]^ The protocol was elaborated and registered in the International Prospective Register of Systematic Reviews (PROSPERO) under the registration number CRD42023399611.

The focused research question was: what are the clinical and histological effects of ATB grafts for LARA compared to ABB grafts?

### 2.2. Selection criteria

The PICOS framework was applied as the eligibility criterion, as shown in Table 1.

**Table 1:** Selection criteria.

Inclusion criterion	Details
Population (P)	Systemically healthy patients with insufficient alveolar ridge width but sufficient height in edentulous regions requiring simultaneous or staged implant placement
Intervention (I)	ATB grafts from the prepared root of the extraction tooth for LARA
Comparison (C)	ABB grafts from intraoral alveolar bone for LARA
Outcomes (O)	Ridge width gain, graft resorption, postoperative complications, histology, and histomorphometry
Study design (S)	Prospective/retrospective cohort studies, clinical or randomized controlled trials
Exclusion criterion	Details
Inappropriate recipient sites	e.g., poor bone quality, fresh extraction sockets, insufficient ridge height, or soft tissue defects
Inappropriate surgical protocols	e.g., alveolar ridge preservation, maxillary sinus floor augmentation, vertical ridge augmentation, grafted with root/bone particles, or non-autogenous root/bone blocks
Inappropriate study design	e.g., conference abstracts, reviews, case (series) reports, in vitro studies, or animal studies
Published in a language other than English and Chinese	
The follow-up time was <3 mo, or participants were <5	

ABB = autogenous bone block, ATB = autogenous tooth block, LARA = lateral alveolar ridge augmentation.

### 2.3. Search strategy

Two authors (Z.R.M. and L.J.X.) searched for relevant studies in the MEDLINE, Embase, Cochrane Library (CENTRAL), Scopus, Web of Science, China national knowledge infrastructure, Wanfang data, and Sinomed databases by using the specified search strategy shown in Table 2. Databases were searched from the date of their inception to July 18, 2023. Furthermore, a manual search of professional oral implantology and surgery journals from January 2000 to July 2023 was performed to locate any potentially eligible studies, including *Journal of Clinical Periodontology, Journal of Periodontology, Clinical Oral Implants Research, Clinical Implant Dentistry and Related Research, Clinical oral investigation, International Journal of Oral and Maxillofacial Implants, International Journal of Oral Implantology, Implant Dentistry, International Journal of Implant Dentistry, Journal of Periodontal and Implant Science, and Journal of Oral Implantology*.

**Table 2:** The search strategy used for each database.

Database	Search strategy	Records
MEDLINE	((((((Alveolar Ridge Augmentation[MeSH Terms]) OR (lateral ridge augmentation[Title/Abstract])) OR (bone augmentation[Title/Abstract])) OR (ridge reconstruction[Title/Abstract])) OR (horizontal ridge augmentation[Title/Abstract]))) AND ((((((Tooth[MeSH Terms]) OR (root graft[Title/Abstract])) OR (autogenous tooth[Title/Abstract])) OR (tooth root[Title/Abstract])) OR (tooth root graft[Title/Abstract])) OR (dentin[Title/Abstract])) Filters: Clinical Trial, Randomized Controlled Trial, Humans	45
Embase	(“root graft” OR “autogenous tooth” OR “tooth root”/exp OR “tooth root” OR “tooth root graft” OR “dentin”/exp OR “dentin”) AND (“alveolar ridge augmentation”/exp OR “alveolar ridge augmentation” OR “lateral ridge augmentation” OR “bone augmentation”/exp OR “bone augmentation” OR “ridge reconstruction” OR “horizontal ridge augmentation”) AND “human”/de AND “article”/it	74
Cochrane library	(“root graft” OR “autogenous tooth” OR “tooth root” OR “tooth root graft” OR “dentin”) AND (“alveolar ridge augmentation” OR “lateral ridge augmentation” OR “bone augmentation” OR “ridge reconstruction” OR “horizontal ridge augmentation”) in Title Abstract	26
Scopus	TITLE-ABS-KEY ((“root graft” OR “autogenous tooth” OR “tooth root” OR “tooth root graft” OR “dentin” AND “alveolar ridge augmentation” OR “lateral ridge augmentation” OR “bone augmentation” OR “ridge reconstruction” OR “horizontal ridge augmentation”)) AND (LIMIT-TO (DOCTYPE, “ar”)) AND (LIMIT-TO (EXACTKEYWORD, “Human”))	118
Web of science	(“root graft” OR “autogenous tooth” OR “tooth root” OR “tooth root graft” OR “dentin”) AND (“alveolar ridge augmentation” OR “lateral ridge augmentation” OR “bone augmentation” OR “ridge reconstruction” OR “horizontal ridge augmentation”) AND (“Human”) NOT (“Review”)	99
CNKI	Chinese word ((“Alveolar Ridge Augmentation” OR “lateral ridge augmentation” OR “horizontal ridge augmentation”)) AND ((“autogenous tooth” OR “ dentin” OR “dentin block”))	33
Wanfang data	Chinese word ((“Alveolar Ridge Augmentation” OR “lateral ridge augmentation” OR “horizontal ridge augmentation”)) AND ((“autogenous tooth” OR “ dentin” OR “dentin block”))	84
SinoMed	Chinese word ((“Alveolar Ridge Augmentation” OR “lateral ridge augmentation” OR “horizontal ridge augmentation”)) AND ((“autogenous tooth” OR “ dentin” OR “dentin block”))	3

CNKI = China national knowledge infrastructure.

### 2.4. Screening and data collection

After the initial publications were retrieved, 2 independent reviewers (Z.R.M. and L.J.X.) performed sequential screening, deleted repeated studies, read the title and abstract, reviewed the full text, and determined their eligibility for inclusion. The agreement between the reviewers was assessed by calculating Cohen kappa (using GraphPad online: https://www.graphpad.com/quickcalcs/kappa1.cfm).

The following data were recorded: author, year of publication, study design, number of patients and implants, age, origin of grafted blocks, characteristics of recipient sites, process of grafted blocks, implant placement period, follow-up period, and outcome. Subsequently, unmatched information was crosschecked and verified to exclude studies that did not meet the inclusion criteria. Incomplete or unclear information concerning the requirements was supplemented by contacting the corresponding author via email.

### 2.5. Outcome variables

Ridge width gain and graft resorption were set as the primary outcome variables, and postoperative complications, histology, and histomorphometry were set as the secondary outcome variables. For comparability of results, several quantitative outcome variables were defined as follows: Ridge width gain was measured as the change in buccal-lingual width at the level of the proximal alveolar ridge from preoperative to reentry. Graft resorption was measured as the change in buccolingual width reduction from immediate grafting to reentry. Postoperative complications were defined as adverse events that occurred during the observation period after grafting.

### 2.6. Quality assessment

Two evaluators (M.N. and G.Y.) independently assessed the methodological quality of the clinical controlled trials (CCTs) according to the standard Newcastle–Ottawa Scale.^[27]^ Eight prominent areas in each study were judged by scoring (the area of comparability scored 2, and the remaining 7 regions scored 1). The highest score in a single study was 9. A score >7 was considered high quality, whereas a score <6 was considered low methodological quality.

The methodological quality of randomized controlled trials (RCTs) was evaluated using the Cochrane Collaboration risk of bias tool,^[28]^ which consists of 7 domains (sequence generation, allocation concealment, blinding of participants and investigators, blinding of outcome assessment, incomplete data outcome, selective outcome reporting, and potential sources of bias). These areas are graded as “high risk,” “low risk,” or “unclear.”

### 2.7. Data synthesis and subgroup analysis

Review Manager 5.4 was used for the statistical analysis. The mean difference (MD) and relative risk were used for continuous and bivariate variables, respectively. Ninety-five percent confidence intervals (CI) were used and a *P* value < .05 was considered to indicate statistical significance. The chi-square test and *I*^2^ test were used to assess inter-study heterogeneity. In the case of low heterogeneity (*P* > .05 or *I*^2^ ≤ 50%), a fixed model was used; otherwise, a random model was used. A qualitative description was used if the data could not be statistically analyzed.

The follow-up time was used as a subgroup for the statistical analysis when the same outcome variables were pooled.

## 3. Results

### 3.1. Study selection

**Figure 2.**
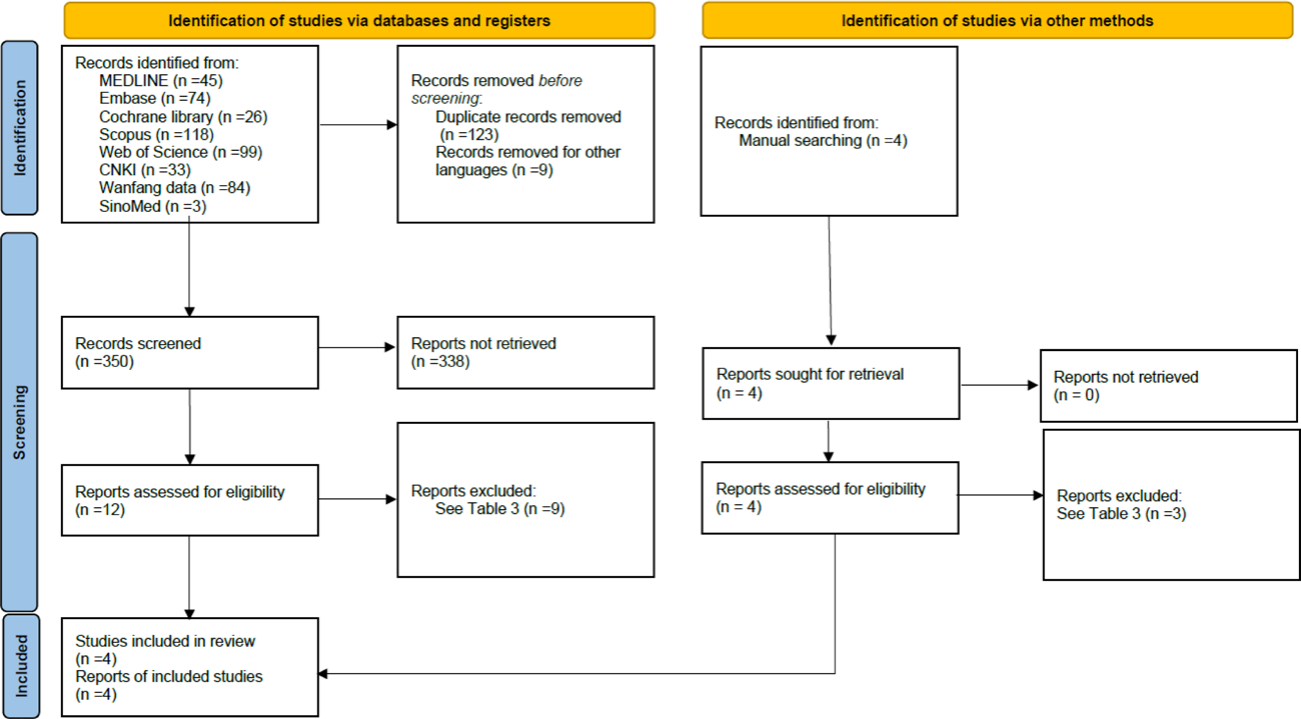
Preferred reporting items for systematic reviews and meta-analyses (PRISMA) flowchart of the search process.

A total of 482 records were identified through electronic searches, including 45 in MEDLINE, 74 in Embase, 26 in the Cochrane Library, 118 in Scopus, 99 in Web of Science, 33 in China national knowledge infrastructure, 84 in Wanfang Data, and 3 in SinoMed. All data were screened, with the initial removal of 123 duplicates and 9 other language articles (non-Chinese or non-English), followed by reviewing of titles and abstracts, resulting in the exclusion of 122 records. The full texts of the remaining 12 articles and 4 retrieved manually were reviewed, and only 1 RCT^[29]^ and 3 CCTs^[30–33]^ were included in this systematic review (Fig. 2). The reasons for the exclusion of articles are listed in Table 3. Moreover, the 2 CCTs^[31,32]^ were confirmed in separate cohorts via email with the corresponding authors. The kappa value was 0.83 for the selection process.

**Table 3:** Studies excluded after full text review.

Excluded studies	Reasons for exclusion
Elraee et al 2022	Conference abstracts
Schwarz et al 2022	Insufficient ridge dimension requiring horizontal and vertical augmentation procedure
Xiao et al 2019	Extraction socket, non-autogenous grafted material
Groβ et al 2022	Inappropriate study design
Kim et al 2013	Case series report
Kim 2015	Extraction socket preservation
Kim et al 2017	Extraction socket preservation
Parvini et al 2020	Extraction socket
Pohl et al 2017	The number of appropriate participants <5
Schwarz et al 2019	Extraction socket
Shejali et al 2019	Extraction socket
Wang et al 2022	Extraction socket

### 3.2. Study characteristics

In a total of 4 studies, 154 patients underwent treatment with 164 implants. Out of these, 77 patients received 77 implants in the ATB group, while 77 patients received 87 implants in the ABB group. Patient age was specified in all studies. The ATB grafts were taken from wisdom and unreserved autogenous teeth, while the ABB grafts were mostly taken from the retromolar region. The edentulous areas with a maximum of 2 missing teeth were found to be deficient in ridge width but had appropriate height and soft tissue conditions. All studies used staged implant placement, except for the study by Korsch et al^[30]^ which used simultaneous implant placement. The follow-up period ranged from 5 months to 26 weeks, at which time the healing process at the recipient sites stabilized (Table 4).

**Table 4:** Characteristics of clinical studies that compared ATB with ABB grafts.

Author/publication yr	Study design	No. patients/implants	Age (yr)	Origins of grafted block	Characteristics of recipient sites	Process of grafted blocks	Implant placement period	Follow-up
Elraee et al 2022	RCT	ATB: 21/21	ATB: 30.1 ± 6.5	ATB: lower wisdom	Single upper central incisor with moderate horizontal defect 4–8 mm (Cologne Classification), sufficient ridge height, at least 3mm keratinized tissue	ATB: the crown, pulp, and cementum were removed to obtain 4mm width root dentin matching the size and shape of the defect area	Staged implantation after 6 mo	6 mo
		ABB: 21/21	ABB: 28.7 ± 4.4	ABB: retromolar area and ascending ramus		ABB: monocortical block was obtained from the retromolar region		
Korsch et al 2021	CCT	ATB: 28/28	ATB: 62 ± 11.4	ATB: non-retainable tooth or wisdom tooth	Lateral ridge defect ≥ 4mm, edentulous region of maximum 2 missing teeth	ATB: the obtained root dentin with a thickness of 1–1.5mm was partially demineralized by removing the crown, cementum, pulp, and filling material	Simultaneous implantation	5 mo
		ABB: 31/41	ABB: 60.4 ± 13.9	ABB: retromolar area of the mandible		ABB: autogenous cortical bone slice through longitudinal splitting of the bone block		
Schwarz et al 2018	CCT	ATB: 15/15	ATB: 41.93 (19∼60)	ATB: wisdom or impacted teeth	Insufficient ridge width, sufficient ridge height, at least 3mm keratinized tissue	ATB: the crown, pulp, and cementum were removed to obtain root dentin matching the size and shape of the defect area	Staged implantation after 26 wk	26 wk
		ABB: 15/15	ABB: 44.53 (21∼60)	ABB: retromolar area		ABB: monocortical block was obtained from the retromolar region		
Schwarz et al 2019	CCT	ATB: 13/13	ATB: 51.23 ± 23.39	ATB: wisdom or impacted teeth	Insufficient ridge width, sufficient ridge height, at least 3mm keratinized tissue	ATB: the crown, pulp, and cementum were removed to obtain root dentin matching the size and shape of the defect area	Staged implantation after 26 wk	26 wk
		ABB: 10/10	ABB: 42.80 ± 13.66	ABB: retromolar area		ABB: monocortical block was obtained from the retromolar region		

Author/publication yr	Ridge width gain (mm)	Graft resorption (mm)	Postoperative complications	Histology	Histomorphometry
Elraee et al 2022	ATB: 3.52 ± 0.56	ATB: 0.48 ± 0.66	ATB: 0	ATB: new bone formation and viable cells and matrix on the dentin block periphery. Newly formed osteoid tissue was deposited in the connection between the dentin block and native bone, which had osteocytes and vessels.	ATB: Bone: 42.6%, fibrous tissue: 6.7%, residual dentin: 31.5%
	ABB: 2.24 ± 0.86	ABB: 1.05 ± 1.43	ABB: 0	ABB: most of the bone was surrounded with fibrous tissue with less evidence of new bone formation in addition to the fibrous tissue connection between native bone and the new bone block.	ABB: Bone: 41.3%, fibrous tissue: 4.6%
Korsch et al 2021			ATB: 1 wound dehiscence		
			ABB: 2 wound dehiscence, 4 inflammations		
Schwarz et al 2018	ATB: 5.53 ± 1.88	ATB: 0.13 ± 0.97	ATB: 1 screw exposure		
	ABB: 3.93 ± 1.41	ABB: 1.03 ± 1.15	ABB: 1 secondary graft, 1 screw exposure		
Schwarz et al 2019	ATB: 5.5 ± 1.73		ATB: 6 peri-implant mucositis		
	ABB: 4.25 ± 1.76		ABB: 2 peri-implant mucositis		

ABB = autogenous bone block, ATB = autogenous tooth block, CCT = clinical controlled trial, RCT = randomized controlled trial.

### 3.3. Quality assessment

In the study by Korsch et al,^[30]^ 1 score was subtracted for comparability owing to the shapes of the grafted blocks. Nevertheless, all CCTs had scores >7 and were considered to be of high quality. The RCT by Elaree et al,^[29]^ was identified as having a high risk of “performance bias” and “blinding of outcome assessment” because of the inability to mask operators and outcome examiners (Table 5).

**Table 5:** Assessment of the risk of bias for the included studies.

Cochrane Collaboration tool for randomized controlled trials	Random sequence generation (selction bias)	Allocation concealment (selection bias)	Blinding of participants and personnel (performance bias)		Blinding of outcome assessment	Incomplete outcome data (attrition bias)	Selective reporting (reporting bias)	Other bias
Elraee et al 2022	Low risk	Low risk	High risk		High risk	Low risk	Low risk	Low risk

Newcastle-Ottawa Scale for controlled clinical trials	Representativeness of the exposed cohort	Selection of the non-exposed cohort	Ascertainment of exposure	Demonstration that outcome of interest was not present at start of study	Comparability of cohorts on the basis of the design or analysis	Assessment of outcome	Was follow-up long enough for outcomes to occur	Adequacy of follow-up of cohorts
Korsch et al 2021	1	1	1	1	1	1	1	1
Schwarz et al 2018	1	1	1	1	2	1	1	1
Schwarz et al 2019	1	1	1	1	2	0	1	1

### 3.4. Synthesis of results

#### 3.4.1. Ridge width gain.

**Figure 3.**
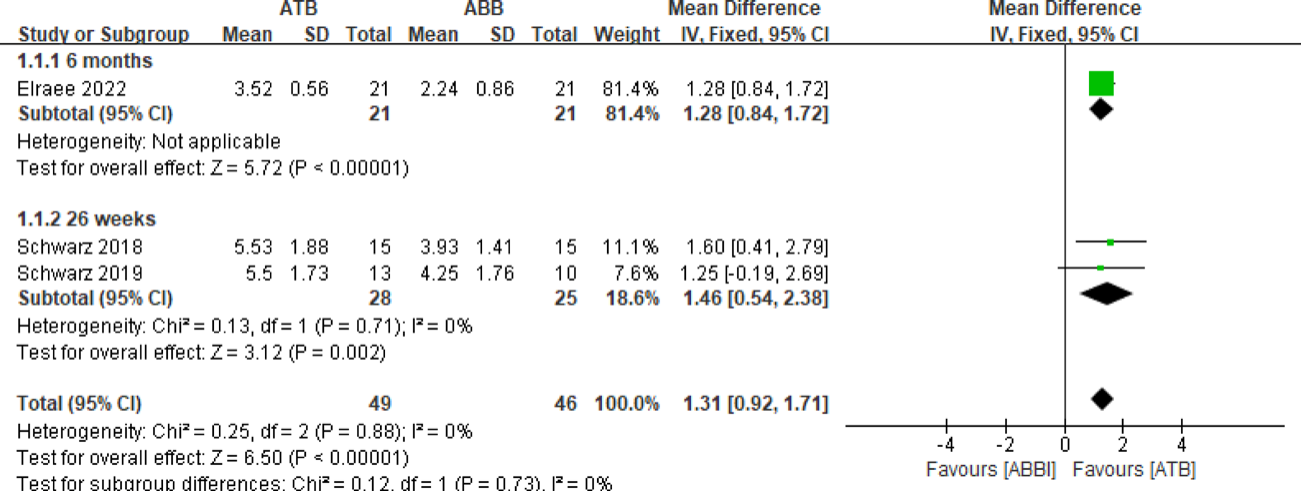
Forest plot of ridge width gain.

Three articles^[29,31,32]^ reported ridge width gain after augmentation surgery. The meta-analysis showed that the ATB group gained more ridge width than the ABB group (MD = 1.31, 95% CI [0.92, 1.71]; fixed-model, *P* < .00001), with no heterogeneity (*I*^2^ = 0%) (Fig. 3).

#### 3.4.2. Graft resorption.

**Figure 4.**
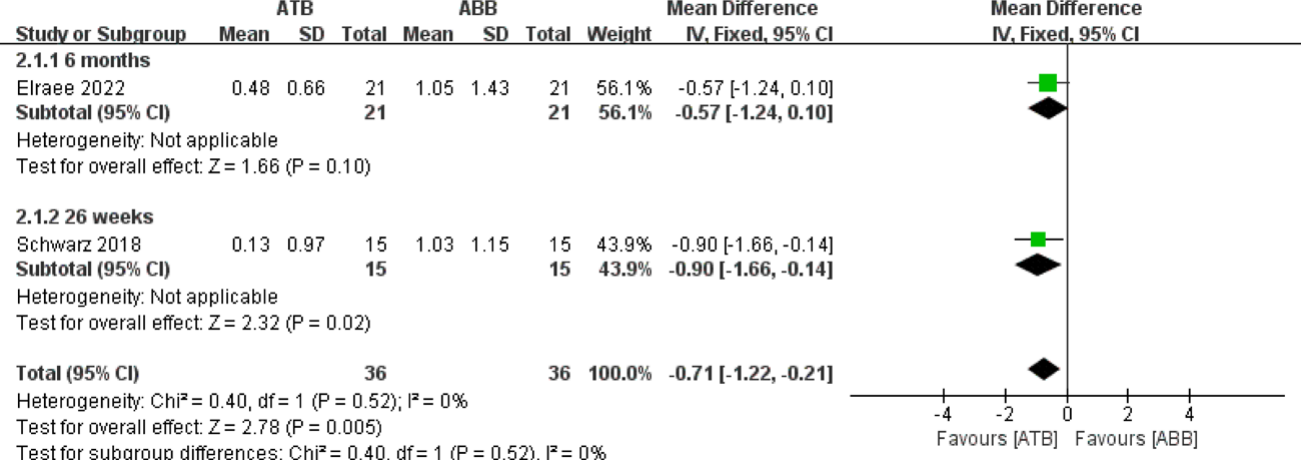
Forest plot of graft resorption.

Two studies^[29,31]^ reported horizontal graft absorption, and the results showed that the ATB group had less graft resorption than the ABB group (MD = −0.71, 95% CI [−1.22, −0.21]; fixed-model, *P* = .005), with no heterogeneity (*I*^2^ = 0%) (Fig. 4).

#### 3.4.3. Postoperative complications.

**Figure 5.**
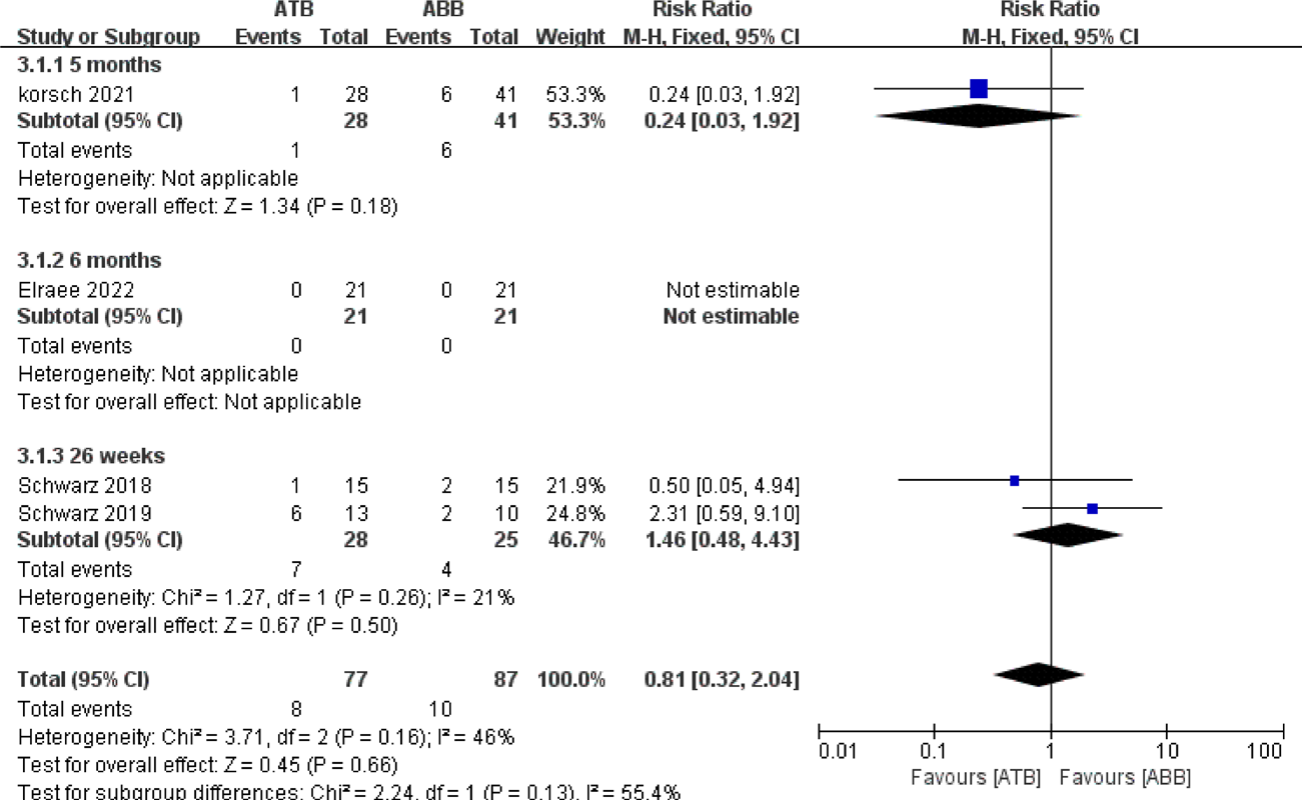
Forest plot of postoperative complications.

All studies^[29–32]^ mentioned the presence of postoperative complications, although 1 study^[29]^ did not report any adverse events. Two studies^[30,31]^ documented 1 wound dehiscence and 1 screw exposure in the ATB group, whereas the ABB group experienced 2 wound dehiscences, 4 inflammations, and 1 insufficient bone gain requiring secondary grafting. In one study,^[32]^ peri-implant complications were observed, with 6 cases of peri-implant mucositis in the ATB group and 2 cases in the ABB group. According to the meta-analysis, there was no significant difference in postoperative complications between the 2 groups (relative risk = 0.81, 95% CI [0.32, 2.04]; fixed-model, *P* = .66), with low heterogeneity (*I*^2^ = 46%) (Fig. 5).

#### 3.4.4. Histology.

During implant bed preparation, Elraee et al^[29]^ obtained core biopsy samples including ATB and ABB. Upon microscopic examination, viable cells and matrix as well as new bone formation, were observed on the periphery of the ATB. The connection between the ATB and native bone also showed the deposition of newly formed osteoid tissue. In contrast, in the ABB group, a fibrous tissue connection was observed between the ABB and native bone.

#### 3.4.5. Histomorphometry.

Elraee et al^[29]^ performed histomorphometric analysis of 200 µm sections and found that the bone area, fibrous tissue area, and residual dentin fraction in the ATB group were 42.6%, 6.7%, and 31.5%, respectively. In the ABB group, the bone area fraction was 41.3% and the fibrous tissue area fraction was 4.6%. No significant differences were detected between the 2 groups (*P* > .05).

## 4. Discussion

The present systematic review involved 154 patients with 77 ATB and 87 ABB grafts and compared the clinical and histological behaviors of ATB and ABB grafts in LARA. Our results contradict the initial assumption that ATB grafts result in greater horizontal ridge width and less graft resorption than ABB grafts on LARA.

Reconstructing bone for non-contained and atrophic ridge defects is particularly challenging and generally recommends the application of volume-stable block grafts.^[3]^ Although substitutes such as allografts, xenografts, and alloplasts are available, they do not offer the same level of effectiveness as ABB grafts in terms of their osteogenic properties.^[34]^ Based on the reviewed studies, cortico-cancellous ABB from the intraoral retromolar area and ascending ramus is the preferred approach, with the cortical side serving as the outer contour and the cancellous side in direct contact with the alveolar bone. While this method can help minimize absorption and promote graft survival, it is essential to note that some patients may experience donor-related complications such as discomfort, infection, and neurosensory disorders.^[35]^

Beginning in the 1960s, Morris et al^[36,37]^ attempted to graft human-derived tooth roots into the subcutaneous tissues of rats and observed surprising bone remodeling. Since then, various animal and human studies have shown promising preliminary results using autogenous particulate roots for guided bone regeneration.^[38,39]^ Following these biological foundations, ATB prepared at the chairside immediately after tooth extraction offers an alternative to ABB, owing to its homogeneous sources, lower morbidity, and less donor discomfort.^[22]^ Nonetheless, this technique is only partially applicable when wisdom or non-retained teeth are available. Ideally, healthy donor teeth are best for ATB grafts; however, autogenous teeth with endodontically treated or periodontitis are also plausible.^[23]^

Based on the findings of this review, using ATB grafts resulted in a greater increase in average bone width (ranging from 3.52 ± 0.56 mm to 5.5 ± 1.73 mm) compared to ABB grafts (ranging from 2.24 ± 0.86 mm to 4.25 ± 1.76 mm). This finding was consistent with those of previous studies,^[40–42]^ which suggested that the positive outcome was due to a more stable environment for bone formation and replacement resorption of the basal bone in the recipient area.^[29,32]^

Considering the limited volume of ATB grafts, the included studies^[29–32]^ limited their investigations to <2-tooth defective regions and mild to moderate horizontal defects.^[42]^ Several studies^[33,41]^ have shown that ATB can be effective in treating severe lateral or combined vertical and horizontal ridge deficiencies; however, they should be used in conjunction with other bone substitutes. With reference to the current protocol of LARA,^[43]^ ATB is a viable alternative to onlay grafting when the buccolingual bone width is between 3 and 6 mm, provided that a sufficient amount is available from non-retained teeth.

The ability of graft materials to resist resorption determines the final efficacy of horizontal bone augmentation.^[29]^ Our study revealed that ATB grafts had less mean horizontal absorption (0.13 ± 0.97 mm ~ 0.48 ± 0.66 mm) compared to ABB grafts (1.03 ± 1.15 mm ~ 1.05 ± 1.43 mm). This result aligns with those of the studies conducted by Kim et al^[13]^ and Parvini et al.^[44]^ There were 2 reasons for this finding. First, the resorption of ATB begins at the basal side where fibrous tissue and woven bone rapidly form new structures towards the dentin after basal ankylosis.^[29,31]^ Second, the high stiffness of dentin prevents superficial root absorption and supports collagen fiber attachment.^[31,33]^ Conversely, ABB grafts without barrier membrane protection may accelerate absorption owing to the occurrence of a substantial amount of non-vital bone regions along with weak vascular tissue.^[45]^

The reported postoperative complications associated with the use of ATB grafts included one instance of wound dehiscence (1.3%), 1 case of fixation screw exposure (1.3%), and peri-implant mucositis in 6 patients (7.8%). However, these events did not cause graft failure after a brief management. The occurrence of peri-implant mucositis may be linked to the lack of chemical cleansing process.^[46]^ A recent study^[44]^ showed satisfactory stability in peri-implant tissue conditions after LARA using ATB grafts, as evidenced by changes in bleeding on probing, probing pocket depth, mucosal recession, and clinical attachment level.

Inflammation was the most common complication associated with ABB grafts (4.6%), followed by wound dehiscence (2.3%), peri-implant mucositis (2.3%) and screw exposure (1.1%). Our results differed slightly from those of a previous study^[40]^ that found soft tissue dehiscence with premature graft exposure to be the most common issue (14.3%). One particularly concerning event required secondary augmentation due to the excessive absorption of ABB grafts.^[31]^ Studies have reported severe adverse events associated with ABB grafting, such as neurosensory disturbance and infection at the donor site,^[47,48]^ which can be avoided using ATB grafts.

While our findings indicate that TB grafts do not have a higher rate of postoperative complications than ABB grafts, the lack of long-term evidence on implant prognosis prevented us from making a definitive conclusion on the superior reliability of ATB compared to ABB grafts.

All simultaneous and staged implants survived in the reviewed studies. Similarly, Bazal-Bonelli et al^[42]^ reported a 98.32% survival rate of implants in various types of alveolar ridge defects with ATB grafts, which was attributed to osseointegration strength. Korsch et al^[30]^ found that the average implant stability quotient in the ATB group (73.3) was similar to that in the ABB group (74.7) 3 months after implant placement. Further animal studies^[22,25]^ showed comparable results in both groups in terms of implant removal torque (ATB:61.00 ± 29.27 Ncm vs ABB:50.92 ± 40.80 Ncm) and bone-implant contact (ATB:50.79% vs ABB:32.53%). Overall, the success rate of titanium implants placed following LARA using ATB grafts was satisfactory and comparable to that of ABB grafts in the short term based on the current level of evidence.

Considering histology, Elraee et al^[29]^ observed that the peripheral compartments of the ATB were invaded and replaced by non-mineralized vascularized woven bone, accompanied by new areas of calcification and matrix formation. The remodeling pattern of the ABB grafts was similar to that of the ATB grafts, with cortical bone undergoing incomplete revascularization and a “creeping substitution process,” but basal cancellous bone underwent faster replacement resorption.^[22,49]^ In a quantitative assessment of the difference in replacement absorption between ATB and ABB grafts in LARA, Parvini et al^[50]^ observed that ATB had higher surface area values (ATB:22.07 ± 12.98 mm^2^ vs ABB:12.42 ± 10.11 mm^2^) but lower basal integration values (ATB:69.26 ± 26.01% vs ABB:79.67 ± 15.66%) compared to ABB. It has been confirmed that the absorption rate of ATB grafts is slower than that of ABB grafts.^[51]^

With regard to histomorphometry, Elraee et al^[29]^ was the only study that reported comparable new bone area proportions for ATB (42.6%) and ABB grafts (41.3%) after 6 months of healing. Published clinical evidence for histomorphometric analysis is scarce, but various preclinical trials have shown favorable results for ATB data, including 66.16 ± 19.64% mineralized tissue^[44]^ and residual grafts ranging from 3.04% to 7.28%.^[24]^ These findings confirm that ATB exhibits good biocompatibility and a slow absorption rate for at least 6 months.^[29]^

Although simultaneous implant placement has been reported as feasible,^[30]^ most scholars still prefer staged implant placement to ensure interference-free healing. This cautious approach may be due to factors such as empirical recognition, type of ridge defect, patient-specific considerations, and volume of ATB.^[29,46]^ Therefore, future clinical studies should focus on determining specifications for chairside preparation of ATB, including optimal morphology, suitable types of ridge defects, and timing of implantation.

The main limitation of this systematic review was the relatively few studies available, which could potentially amplify the bias of individual studies despite the low inter-study heterogeneity. Additionally, the longest follow-up time of the included studies was only 26 weeks, making the long-term performance of the ATB grafts compared to ABB grafts unclear.

## 5. Conclusion

Within the limitations of this study, ATB grafts for LARA resulted in greater bone width and less graft resorption than did ABB grafts. Moreover, ATB grafts are capable of replacement resorption and of stable integration with the alveolar bone. ATB grafts can serve as alternative materials to support LARA in the presence of a non-retainable teeth. Further evidence from long-term RCTs is needed to substantiate the efficacy of ATB grafts in LARA.

## Acknowledgments

Authors are thankful to Mr. Lang Xin for his help with the methodological evaluation.

## Author contributions

**Data curation:** Yan Guo, Jianxue Li.

**Formal analysis:** Yan Guo, Jianxue Li.

**Methodology:** Ruimin Zhao, Jiaming Gong.

**Project administration:** Delin Guan, Jiaming Gong.

**Validation:** Delin Guan, Na Ma.

**Visualization:** Delin Guan, Na Ma.

**Writing – review & editing:** Delin Guan, Jiaming Gong.

**Writing – original draft:** Ruimin Zhao, Jiaming Gong.

## References

[1] Masaki C Nakamoto T Mukaibo T. Strategies for alveolar ridge reconstruction and preservation for implant therapy. J Prosthodont Res. 2015;59:220–8. 26022542 10.1016/j.jpor.2015.04.005

[2] Barone A Ricci M Tonelli P. Tissue changes of extraction sockets in humans: a comparison of spontaneous healing vs. ridge preservation with secondary soft tissue healing. Clin Oral Implants Res. 2013;24:1231–7. 22784417 10.1111/j.1600-0501.2012.02535.x

[3] Elnayef B Porta C Suárez-López Del Amo F. The fate of lateral ridge augmentation: a systematic review and meta-analysis. Int J Oral Maxillofac Implants. 2018;33:622–35. 29763500 10.11607/jomi.6290

[4] Mcallister BS Haghighat K. Bone augmentation techniques. J Periodontol. 2007;78:377–96. 17335361 10.1902/jop.2007.060048

[5] Chiapasco M Casentini P Zaniboni M. Bone augmentation procedures in implant dentistry. Int J Oral Maxillofac Implants. 2009;24(Suppl):237–59. 19885448

[6] Von Arx T Cochran DL Hermann JS. Lateral ridge augmentation using different bone fillers and barrier membrane application. A histologic and histomorphometric pilot study in the canine mandible. Clin Oral Implants Res. 2001;12:260–9. 11359484 10.1034/j.1600-0501.2001.012003260.x

[7] Aloy-Prósper A Peñarrocha-Oltra D Peñarrocha-Diago M. The outcome of intraoral onlay block bone grafts on alveolar ridge augmentations: a systematic review. Med Oral Patol Oral Cir Bucal. 2015;20:e251–258. 25662543 10.4317/medoral.20194 PMC4393991

[8] Chappuis V Cavusoglu Y Buser D. Lateral ridge augmentation using autogenous block grafts and guided bone regeneration: a 10-year prospective case series study. Clin Implant Dent Relat Res. 2017;19:85–96. 27476677 10.1111/cid.12438

[9] Nielsen HB Starch-Jensen T. Lateral ridge augmentation in the posterior part of the mandible with an autogenous bone block graft harvested from the ascending mandibular ramus. A 10-year retrospective study. J Stomatol Oral Maxillofac Surg. 2021;122:141–6. 32480048 10.1016/j.jormas.2020.05.020

[10] Nkenke E Neukam FW. Autogenous bone harvesting and grafting in advanced jaw resorption: morbidity, resorption and implant survival. Eur J Oral Implantol. 2014;7(Suppl 2):S203–17. 24977256

[11] Mckenna GJ Gjengedal H Harkin J. Effect of autogenous bone graft site on dental implant survival and donor site complications: a systematic review and meta-analysis. J Evid Based Dent Pract. 2022;22:101731. 36162883 10.1016/j.jebdp.2022.101731

[12] Nampo T Watahiki J Enomoto A. A new method for alveolar bone repair using extracted teeth for the graft material. J Periodontol. 2010;81:1264–72. 20476887 10.1902/jop.2010.100016

[13] Kim YK Lee J Um IW. Tooth-derived bone graft material. J Korean Assoc Oral Maxillofac Surg. 2013;39:103–11. 24471027 10.5125/jkaoms.2013.39.3.103 PMC3858164

[14] Kim YK Kim SG Oh JS. Analysis of the inorganic component of autogenous tooth bone graft material. J Nanosci Nanotechnol. 2011;11:7442–5. 22103215 10.1166/jnn.2011.4857

[15] Gual-Vaqués P Polis-Yanes C Estrugo-Devesa A. Autogenous teeth used for bone grafting: a systematic review. Med Oral Patol Oral Cir Bucal. 2018;23:e112–9. 29274156 10.4317/medoral.22197 PMC5822533

[16] Avery SJ Sadaghiani L Sloan AJ. Analysing the bioactive makeup of demineralised dentine matrix on bone marrow mesenchymal stem cells for enhanced bone repair. Eur Cell Mater. 2017;34:1–14. 28692113 10.22203/eCM.v034a01

[17] Murata M Sato D Hino J. Acid-insoluble human dentin as carrier material for recombinant human BMP-2. J Biomed Mater Res A. 2012;100:571–7. 22213638 10.1002/jbm.a.33236

[18] Bertassoni LE. Dentin on the nanoscale: hierarchical organization, mechanical behavior and bioinspired engineering. Dent Mater. 2017;33:637–49. 28416222 10.1016/j.dental.2017.03.008 PMC5481168

[19] Jun SH Ahn JS Lee JI. A prospective study on the effectiveness of newly developed autogenous tooth bone graft material for sinus bone graft procedure. J Adv Prosthodont. 2014;6:528–38. 25551014 10.4047/jap.2014.6.6.528 PMC4279053

[20] Yüceer-Çetiner E Özkan N Önger ME. Effect of autogenous dentin graft on new bone formation. J Craniofac Surg. 2021;32:1354–60. 33405453 10.1097/SCS.0000000000007403

[21] Pohl V Schuh C Fischer MB. A new method using autogenous impacted third molars for sinus augmentation to enhance implant treatment: case series with preliminary results of an open, prospective longitudinal study. Int J Oral Maxillofac Implants. 2016;31:622–30. 27183071 10.11607/jomi.4172

[22] Schwarz F Golubovic V Becker K. Extracted tooth roots used for lateral alveolar ridge augmentation: a proof-of-concept study. J Clin Periodontol. 2016;43:345–53. 26580310 10.1111/jcpe.12481

[23] Becker K Jandik K Stauber M. Microstructural volumetric analysis of lateral ridge augmentation using differently conditioned tooth roots. Clin Oral Investig. 2019;23:3063–71. 10.1007/s00784-018-2723-4 30413950

[24] Schwarz F Golubovic V Mihatovic I. Periodontally diseased tooth roots used for lateral alveolar ridge augmentation. A proof-of-concept study. J Clin Periodontol. 2016;43:797–803. 27169909 10.1111/jcpe.12579

[25] Becker K Drescher D Hönscheid R. Biomechanical, micro-computed tomographic and immunohistochemical analysis of early osseous integration at titanium implants placed following lateral ridge augmentation using extracted tooth roots. Clin Oral Implants Res. 2017;28:334–40. 27028526 10.1111/clr.12803

[26] Liberati A Altman DG Tetzlaff J. The PRISMA statement for reporting systematic reviews and meta-analyses of studies that evaluate healthcare interventions: explanation and elaboration. BMJ. 2009;339:b2700. 19622552 10.1136/bmj.b2700 PMC2714672

[27] Wells G A Wells G Shea B. The Newcastle-Ottawa Scale (NOS) for assessing the quality of nonrandomised studies in meta-analyses. 2014.

[28] Higgins JP Altman DG Gøtzsche PC. The Cochrane Collaboration’s tool for assessing risk of bias in randomised trials. BMJ. 2011;343:d5928. 22008217 10.1136/bmj.d5928 PMC3196245

[29] Elraee L Abdel Gaber HK Elsayed HH. Autogenous dentin block versus bone block for horizontal alveolar ridge augmentation and staged implant placement: a randomized controlled clinical trial including histologic assessment. Clin Oral Implants Res. 2022;33:723–34. 35509125 10.1111/clr.13936

[30] Korsch M Peichl M. Retrospective study: lateral ridge augmentation using autogenous dentin: tooth-shell technique vs. bone-shell technique. Int J Environ Res Public Health. 2021;18:3174. 33808616 10.3390/ijerph18063174 PMC8003557

[31] Schwarz F Hazar D Becker K. Efficacy of autogenous tooth roots for lateral alveolar ridge augmentation and staged implant placement. A prospective controlled clinical study. J Clin Periodontol. 2018;45:996–1004. 29972245 10.1111/jcpe.12977

[32] Schwarz F Hazar D Becker K. Short-term outcomes of staged lateral alveolar ridge augmentation using autogenous tooth roots. A prospective controlled clinical study. J Clin Periodontol. 2019;46:969–76. 31241784 10.1111/jcpe.13161

[33] Xiao W Hu C Chu C. Autogenous dentin shell grafts versus bone shell grafts for alveolar ridge reconstruction: a novel technique with preliminary results of a prospective clinical study. Int J Periodontics Restorative Dent. 2019;39:885–93. 31613951 10.11607/prd.4344

[34] Tolstunov L E HJF Broumand V. Bone augmentation techniques for horizontal and vertical alveolar ridge deficiency in oral implantology. Oral Maxillofac Surg Clin North Am. 2019;31:163–91. 30947846 10.1016/j.coms.2019.01.005

[35] Bianchi A Felice P Lizio G. Alveolar distraction osteogenesis versus inlay bone grafting in posterior mandibular atrophy: a prospective study. Oral Surg Oral Med Oral Pathol Oral Radiol Endod. 2008;105:282–92. 18280960 10.1016/j.tripleo.2007.07.009

[36] Morris ML. The implantation of human dentin and cementum into the subcutaneous tissues of the rat. Periodontics. 1967;5:113–22. 4226687

[37] Morris ML. The implantation of human dentin and cementum with autogenous bone into the subcutaneous tissues of the rat. J Periodontol. 1971;42:286–92. 4930229 10.1902/jop.1971.42.5.286

[38] Wu D Zhou L Lin J. Immediate implant placement in anterior teeth with grafting material of autogenous tooth bone vs xenogenic bone. BMC Oral Health. 2019;19:266. 31791302 10.1186/s12903-019-0970-7 PMC6889614

[39] Gomes MF Dos Anjos MJ Nogueira TO. Histologic evaluation of the osteoinductive property of autogenous demineralized dentin matrix on surgical bone defects in rabbit skulls using human amniotic membrane for guided bone regeneration. Int J Oral Maxillofac Implants. 2001;16:563–71. 11516004

[40] Ramanauskaite A Sahin D Sader R. Efficacy of autogenous teeth for the reconstruction of alveolar ridge deficiencies: a systematic review. Clin Oral Investig. 2019;23:4263–87. 10.1007/s00784-019-02869-1 30859329

[41] Wang W Jiang Y Wang D. Clinical efficacy of autogenous dentin grafts with guided bone regeneration for horizontal ridge augmentation: a prospective observational study. Int J Oral Maxillofac Surg. 2022;51:837–43. 34924269 10.1016/j.ijom.2021.06.012

[42] Bazal-Bonelli S Sánchez-Labrador L Cortés-Bretón Brinkmann J. Clinical performance of tooth root blocks for alveolar ridge reconstruction. Int J Oral Maxillofac Surg. 2022;51:680–9. 34507879 10.1016/j.ijom.2021.08.019

[43] Yu SH Wang HL. An updated decision tree for horizontal ridge augmentation: a narrative review. Int J Periodontics Restorative Dent. 2022;42:341–9. 35472110 10.11607/prd.5031

[44] Parvini P Schliephake C Al-Maawi S. Histomorphometrical assessment of vertical alveolar ridge augmentation using extracted tooth roots in the canine. Clin Oral Investig. 2020;24:317–23. 10.1007/s00784-019-02960-7 31102042

[45] Acocella A Bertolai R Colafranceschi M. Clinical, histological and histomorphometric evaluation of the healing of mandibular ramus bone block grafts for alveolar ridge augmentation before implant placement. J Craniomaxillofac Surg. 2010;38:222–30. 19648020 10.1016/j.jcms.2009.07.004

[46] Mahardawi B Rochanavibhata S Jiaranuchart S. Autogenous tooth bone graft material prepared chairside and its clinical applications: a systematic review. Int J Oral Maxillofac Surg. 2023;52:132–41. 35618639 10.1016/j.ijom.2022.04.018

[47] Clavero J Lundgren S. Ramus or chin grafts for maxillary sinus inlay and local onlay augmentation: comparison of donor site morbidity and complications. Clin Implant Dent Relat Res. 2003;5:154–60. 14575631 10.1111/j.1708-8208.2003.tb00197.x

[48] Von Arx T Häfliger J Chappuis V. Neurosensory disturbances following bone harvesting in the symphysis: a prospective clinical study. Clin Oral Implants Res. 2005;16:432–9. 16117767 10.1111/j.1600-0501.2005.01138.x

[49] Qin X Raj RM Liao XF. Using rigidly fixed autogenous tooth graft to repair bone defect: an animal model. Dent Traumatol. 2014;30:380–4. 24597718 10.1111/edt.12101

[50] Parvini P Sader R Sahin D. Radiographic outcomes following lateral alveolar ridge augmentation using autogenous tooth roots. Int J Implant Dent. 2018;4:31. 30264332 10.1186/s40729-018-0142-6 PMC6160378

[51] Minetti E Corbella S Taschieri S. Tooth as graft material: histologic study. Clin Implant Dent Relat Res. 2022;24:488–96. 35507503 10.1111/cid.13097 PMC9544007

